# Stratification by Non-invasive Biomarkers of Non-alcoholic Fatty Liver Disease in Children

**DOI:** 10.3389/fped.2022.846273

**Published:** 2022-04-04

**Authors:** Yiyoung Kwon, Eun Sil Kim, Yon Ho Choe, Mi Jin Kim

**Affiliations:** Department of Pediatrics, Samsung Medical Center, Sungkyunkwan University School of Medicine, Seoul, South Korea

**Keywords:** non-alcoholic fatty liver disease (NAFLD), liver steatosis, liver steatohepatitis, liver fibrosis, children

## Abstract

**Background:**

The spectrum of non-alcoholic fatty liver disease (NAFLD) ranges from isolated hepatic steatosis to non-alcoholic steatohepatitis to fibrosis. We aimed to introduce useful biomarkers released during liver inflammation and fibrogenesis that are easy to use in outpatient clinic and adjust to children to evaluate each NAFLD stage without biopsy.

**Methods:**

This prospective study included 60 patients aged under 19 years whose alanine aminotransferase (ALT) levels were elevated from March 2021. All patients were proven to have NAFLD by ultrasonography and laboratory work-up to exclude other causes of hepatitis. Fibroscan and additional laboratory tests for biomarkers [procollagen type1 amino-terminal propeptide (P1NP), osteocalcin, interleukin-6 (IL-6), and Mac-2 binding protein glycosylated isomer (M2BPGi)] were performed. Fibroscan-AST (FAST) score was used for the comparison of steatohepatitis and liver stiffness measurement (kPa) was used for the comparison of advanced fibrosis.

**Results:**

The biomarker that showed a significant difference between the FAST-positive and negative groups was the P1NP/osteocalcin ratio with a *p*-value of 0.008. The area under receiver operating characteristic (AUROC) of P1NP/osteocalcin ratio^*^ALT values (values obtained through multivariate analysis) was 0.939 with the cut-off value of 305.38. The biomarkers that showed a significant difference between the LSM-positive and negative groups were IL-6 and M2BPGi with a *p*-values of 0.005 and <0.001. AUROC of IL-6 ^*^AST values (values obtained through multivariate analysis) was 0.821 with the cut-off value of 228.15. M2BPGi showed a significant linear relationship with LSM in Pearson correlation analysis (Pearson correlation coefficient = 0.382; *p* = 0.003). The diagnostic capability of M2BPGi to evaluate advanced fibrosis showed an acceptable result (AUROC = 0.742; *p* = 0.022).

**Conclusions:**

Non-invasive biomarkers can be used to predict each stage of NAFLD in children. The measurements of P1NP, IL-6 or M2BPGi along with the basic chemistry tests would help determine the stage of NAFLD they correspond to at the time of initial diagnosis and predict responsiveness after the treatment.

## Introduction

The non-alcoholic fatty liver disease (NAFLD) is asymptomatic to the most patients and discovered incidentally from laboratory examinations due to the elevated liver enzymes. Therefore, the disease is sometimes found after progression. The spectrum of NAFLD according to the progression of the disease is from isolated hepatic steatosis to non-alcoholic steatohepatitis to fibrosis to cirrhosis (1). Although the definitive test for diagnosis of NAFLD and its stage is liver biopsy, several studies have introduced non-invasive imaging techniques and scoring systems to assess the stage of NAFLD (2–6). With the aid of advances in imaging and scoring systems, we are one step closer to assessment of the NAFLD stage without liver biopsy.

Moving forward with these imaging modalities and scoring systems, several studies have begun exploring novel biomarkers that are more convenient for the assessment of the stage of NAFLD. Notably, the aminotransferase levels may fluctuate over time and sometimes be normal when they are followed-up. Unfortunately, normalized aminotransferase levels do not exclude the progression to fibrosis or cirrhosis (7). Representatively, Mac-2 binding protein glycosylated isomer (M2BPGi) is a hematological biomarker that predicts high-grade fibrosis and cirrhosis (8, 9). Besides that, the extracellular matrix (ECM) component, hyaluronic acid, amino-terminal peptide of type III collagen, and tissue inhibitor of metalloproteinase-1 are the introduced biomarkers to predict fibrosis (10, 11).

All the studies on these biomarkers were conducted in adult patients with NAFLD and focused only on the evaluation of advanced fibrosis in the NAFLD stage. Additionally, unlike adults, pediatric patients develop steatohepatitis rather than progress to cirrhosis. Therefore, the research on biomarkers in children should be focused on the stages of steatohepatitis and fibrogenesis. In addition, recent studies have found an increased risk of cardiovascular disease in pediatric patients with NAFLD (12, 13). Although it can be subclinical at young age, atherosclerosis and cardiac dysfunction may occur (12). Therefore, it is an important factor to evaluate metabolic (dysfunction) associated fatty liver disease (MAFLD) in patients with confirmed liver steatosis, suggesting that it is important to detect NAFLD at an early stage in children (14, 15).

When inflammation occurs in liver cells, inflammatory cytokines, such as TNF-alpha, interleukin-6 (IL-6), and IGF are generated. Hepatic stellate cells are also converted to myofibroblasts, and collagen, glycoproteins, and proteoglycans are formed as ECM. This study aims to introduce useful biomarkers released during liver inflammation and fibrogenesis, which are easy to use in the outpatient clinic and to adjust to children to evaluate each stage of NAFLD without biopsy.

## Subjects and Methods

### Patients and Study Design

**Figure 1:**
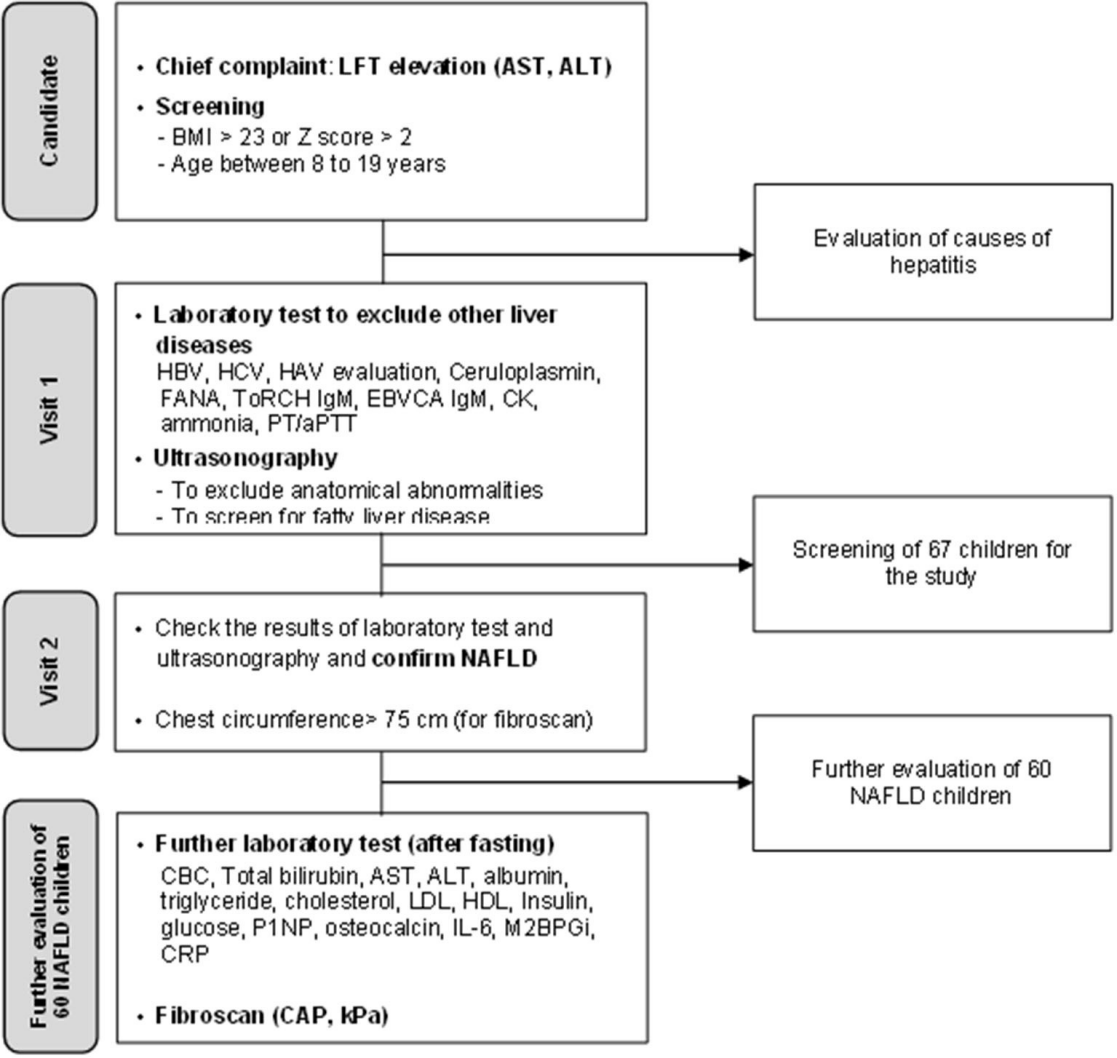
A flowchart illustrating the screening process for non-alcoholic fatty liver disease and enrollment process in this study.

We prospectively enrolled 60 pediatric patients with NAFLD (age <19 years). The initial screening for the study enrollment included patients who visited the out-patient clinic with the chief complaint of elevated alanine aminotransferase (ALT) levels and the body mass index (BMI) >23.0 (Figure 1). They were also screened with questions, such as previous history of inborn errors of metabolism, other liver diseases and whether or not the patients had been administered drugs metabolized by the liver, and showed no signs of infection when they underwent laboratory testing. The patients who met the criteria underwent laboratory tests to exclude other causes of hepatitis and metabolic disease leading to hepatic steatosis as follows: Hepatitis A, B, and C virus, Epstein–Barr virus, levels of creatinine kinase, ceruloplasmin, fasting glucose, ammonia, and fluorescent antinuclear antibody. They also underwent ultrasonography to check for the presence of focal lesion or anatomical abnormalities in the hepatobiliary system and simultaneously check for the presence of fatty liver. Among patients for whom other causes of hepatitis and fatty liver were excluded, ultrasonography screening was used for confirmation; patients with a chest circumference of 75 cm or more were enrolled in the study as a condition for fibroscan examination. Of the 67 patients who were initially recruited, 7 patients dropped out because their chest circumference was <75 cm. Finally, total of 60 patients were included in the study. These 60 patients underwent additional blood tests including biomarkers after fasting and fibroscan to evaluate the stage of NAFLD (Figure 1).

The purpose of this study was to evaluate the diagnostic value of the two measured biomarkers: IL-6 (inflammatory cytokine) and P1NP (ECM product) to assess each stage of NAFLD. The significant differences were compared in patients with steatosis by classifying them into three groups according to their grade of fatty liver on ultrasonography. To evaluate the significant difference in patients with steatohepatitis (in the process of fibrogenesis), we divided the positive and the negative group with the fibroscan-AST (FAST) score and compared them. Advanced fibrosis was compared by dividing the groups based on the liver stiffness measurement (LSM) value obtained through fibroscan.

The second objective of this study was to find out their cut-off values to find meaningful biomarkers. The third objective of this study was to evaluate whether M2BPGi has the diagnostic capability to assess NAFLD with advanced fibrosis in children. All the methods of this study were performed in accordance with the relevant guidelines and regulations and were approved by the Clinical Research Ethics Committee of Samsung Medical Center. We have undergone appropriate written informed consent procedures for analysis of clinical data (IRB File No: SMC 2021-04-064).

### Clinical and Laboratory Data

Clinical data, such as age, sex, and weight (kg) were recorded. BMI was recorded as z-score because the patients were children. Venous blood samples were obtained after fasting over 8 h. The biochemical variables were measured using automated analyzer at the laboratory in the hospital.

We recorded the serum levels for the following: total bilirubin, aspartate aminotransferase (AST), ALT, triglyceride, cholesterol, low density lipoprotein, albumin, platelets, fasting plasma glucose, insulin, c-reactive protein, IL- 6, P1NP, osteocalcin, and M2BPGi.

The values of serum total procollagen type 1 amino-terminal propeptide (P1NP), osteocalcin, and IL-6 were measured using chemiluminescence enzyme immunoassay with the products of Roche Elecsys^®^ (Roche, Basel, Switzerland). P1NP is a representative bone formation marker and is used in the treatment of osteoporosis patients (16). In children and adolescents with rapid bone formation, the standard of normal values is different and higher than that of adults. As the bone formation is rapid, the normal level of P1NP is higher in children and adolescents than in adults. Data regarding osteocalcin, another bone formation marker, were also collected to correct the P1NP values (17). Therefore, in this paper, the value obtained by dividing P1NP by osteocalcin was used as a variable in analysis. In addition, since ALP is a test that can obtain values more easily than osteocalcin, P1NP corrected by ALP was also evaluated. The serum M2BPGi level was measured using chemiluminescence enzyme immunoassay with the HISCL™ M2BPGi™ reagent kit (Sysmex, Kobe, Japan).

### Scoring and Index

The presence of impaired fasting glycemia or diabetes was evaluated by a homeostatic model assessment for insulin resistance, which was calculated as follows: fasting insulin (microU/L) ^*^ fasting glucose (nmol/L)/22.5. A homeostatic model assessment for insulin resistance value of 3.8 or higher was considered as insulin resistance (18, 19). The FAST score, which is the only non-invasive scoring system to evaluate steatohepatitis status, was calculated as follows: e^−1.65+1.07 × *In*(*LSM*)+2.6^6^^*^10^−^8 × *CAP*^3^−63.3 × *AST*^−^1^/1+*e*^−1.65+1.07 × *In*(*LSM*)+2.6^6^^^*^10^−^8 × *CAP*^3^−63.3 × *AST*^−^^1^.

The FAST score of 0.35 or higher is considered as steatohepatitis (20). The fibrosis-4 index is a representative scoring system evaluating advanced fibrosis in NAFLD and is calculated as follows: age (years)^*^AST (U/L)/platelet count (^*^10^9^/L)${}^{*}\sqrt{ALT}$ (U/L) (21).

### Ultrasonography and Fibroscan

The three trained radiology professors evaluated the degree of fatty liver according to the following four grades: (1) grade 0 (absence of steatosis, with normal liver echogenicity); (2) grade 1 (mild steatosis, the liver had a higher echogenicity than the right renal cortex; however, the echogenic wall of the main portal vein was preserved); (3) grade 2 (moderate steatosis, impaired echogenicity of the main portal vein wall); and (4) grade 3 (severe steatosis, impaired visualization of the posterior hepatic parenchyma or the diaphragm) (22, 23). The presence of hepatomegaly and splenomegaly was also evaluated.

Fibroscan (Echosens, Paris, France) was performed by trained nurses. The machine model of fibroscan in the center was 502 TOUCH, with M and XL probes. An automatic probe selection tool was embedded in the software; however, most patients (54/60, 90.0%) were tested using the M probe because most patients were aged under 18 years. The LSM and controlled attenuation parameter (CAP) were evaluated using vibration controlled transient elastography technology. LSM measures the speed of shear wave and expresses the degree of liver fibrosis numerically (unit: kPa). CAP measures the amount of attenuation of ultrasound transmitted into the liver and expresses the degree of fat distribution in the liver numerically (unit: dB/m). Eddewes et al. (24) presented the cut-off values of LSM and CAP in patients with NAFLD through a comparison with biopsy. For LSM, more than 8.2 kPa was evaluated as fibrosis stage 2 or higher (advanced fibrosis), and for CAP, more than 302 kPa was evaluated as steatosis stage 1 or higher (24).

### Statistical Analysis

**Figure 2:**
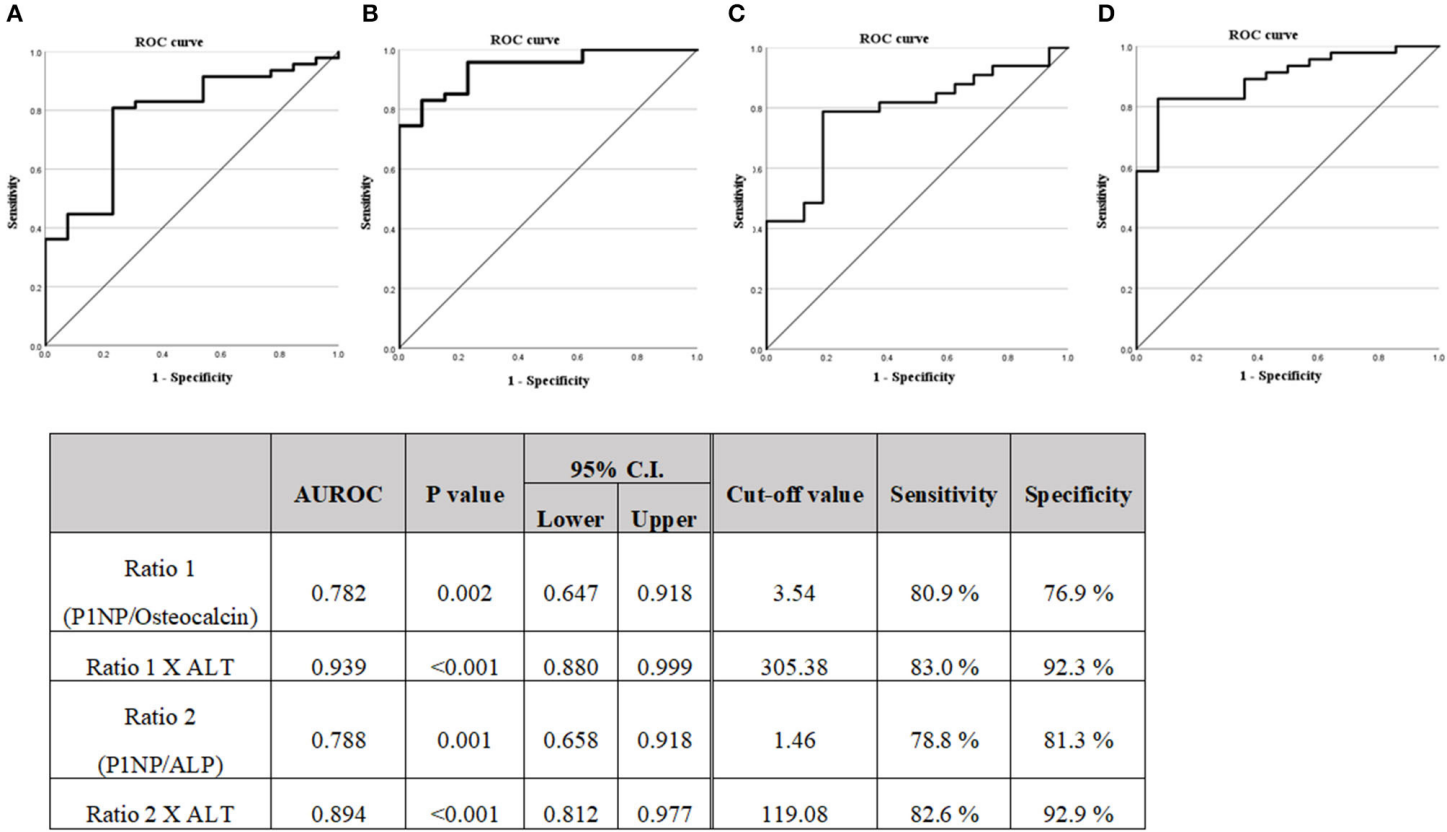
**(A)** Diagnostic capabilities with the area under the receiver operating characteristic curve of the serum P1NP/Osteocalcin ratio values for assessing the steatohepatitis. **(B)** Diagnostic capabilities with the area under the receiver operating characteristic curve of the serum P1NP/Osteocalcin ratio X ALT values for assessing steatohepatitis. **(C)** Diagnostic capabilities with the area under the receiver operating characteristic curve of the serum P1NP/ALP ratio values for assessing steatohepatitis. **(D)** Diagnostic capabilities with the area under the receiver operating characteristic curve of the serum P1NP/ALP ratio X ALT values for assessing steatohepatitis. P1NP, Procollagen type 1 N-terminal propeptide.

**Figure 3:**
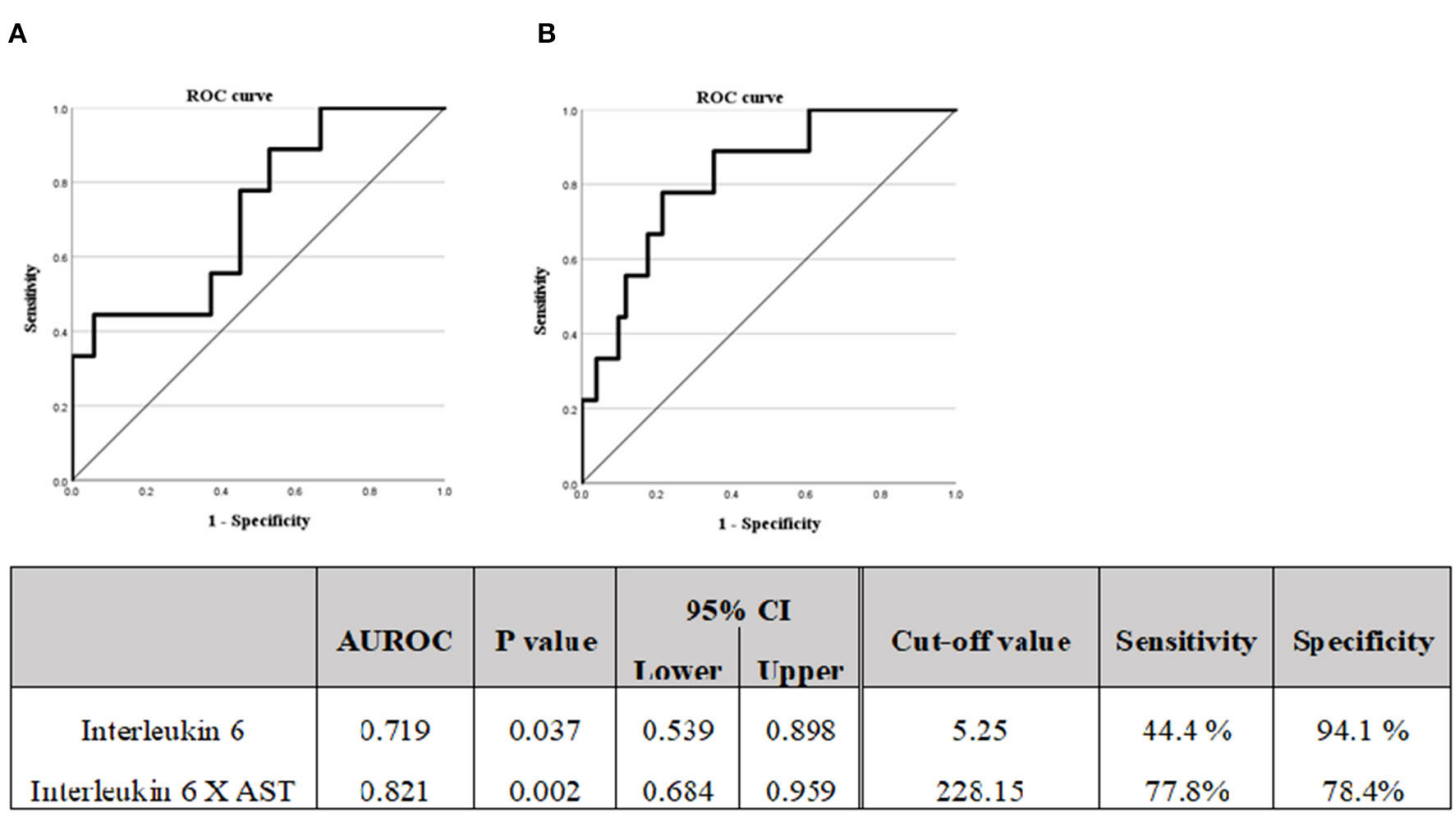
**(A)** Diagnostic capabilities with the area under the receiver operating characteristic curve of the serum interleukin 6 values for assessing the advanced liver fibrosis. **(B)** Diagnostic capabilities with the area under the receiver operating characteristic curve of the serum Interleukin 6 X AST values for assessing advanced liver fibrosis. M2BPGi, Mac-2 binding glycosylation.

**Figure 4:**
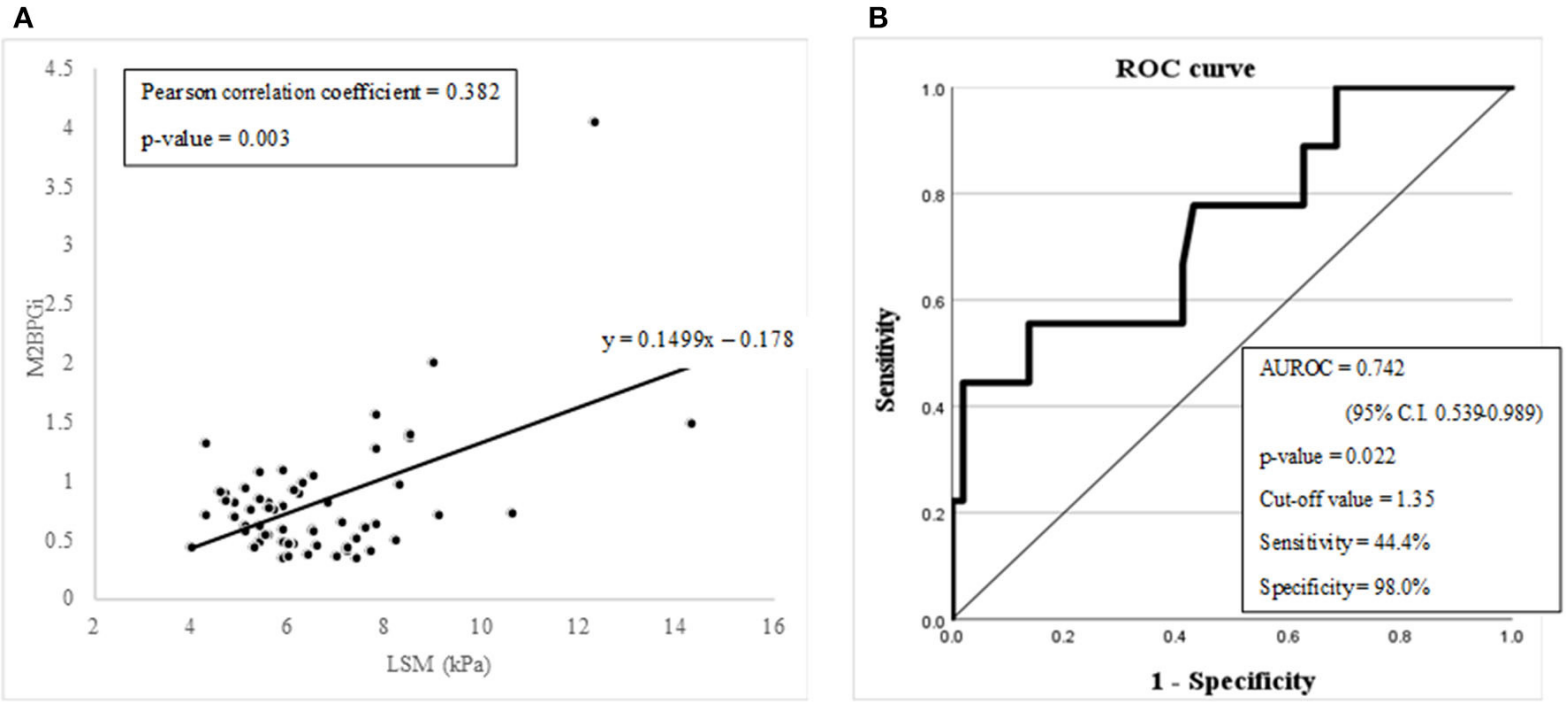
**(A)** A graph showing the linear correlation between M2BPGi and LSM (kPa) with Pearson correlation analysis. **(B)** Diagnostic capabilities with the area under the receiver operating characteristic curve of the serum M2BPGi values for assessing advanced liver fibrosis.

For descriptive statistics, the continuous variables were expressed as mean (standard deviation) and categorical variables were expressed as an absolute number with percentages. In Table 1, one-way analysis of variance test was performed to analyze the mean value comparison between the three groups classified according to the fatty liver grades of ultrasonography. In Table 2, the *t*-test and the Mann–Whitney test were performed to analyze the mean value comparison between the two groups classified as positive and negative for the FAST score. In Table 3, the *t*-test and the Mann–Whitney test were performed to analyze the mean value comparison between the two groups classified as positive and negative for LSM (kPa) values. Univariable and multivariable analyses of the associations between steatohepatitis or fibrosis and other factors were performed using a logistic regression model (Table 3). Multivariable analysis was performed by selecting variables with *p* < 0.1 in univariate analysis. The diagnostic capabilities were evaluated using the receiver operating characteristic (ROC) curve using the items evaluated based on the results of the logistic regression analysis (Figures 2, 3). The diagnostic performances of the biomarkers were expressed as the diagnostic sensitivity, specificity, and area under the ROC (AUROC) curve. Youden's J statistic was computed to identify the cut-off values. In the analysis to evaluate the correlation between LSM and M2BPGi, the Pearson correlation analysis was used (Figure 4).

**Table 1:** Comparison of the clinical characteristics, laboratory results, and biomarkers released during liver inflammation and fibrogenesis in the patients' groups divided according to the grade of ultrasonography.

	**USG^†^ grade 1 (mild)**	**USG grade 2 (moderate)**	**USG grade 3 (severe)**	***P*** **-value**
	***N*** **(percentage) or mean** **±SD**			
Patient number	10 (16.7%)	31 (51.7%)	19 (31.7%)	
Age	14.31 ± 3.03	13.17 ± 2.74	14.63 ± 2.83	0.184^a^
Male/female	7 (70.0%)/3 (30.0%)	25 (80.6%)/6 (19.4%)	17 (89.5%)/2 (10.5%)	0.506^a^
BMI z score	3.54 ± 1.58	3.37 ± 1.31	3.26 ± 1.36	0.866^a^
Total bilirubin (mg/dL)	0.58 ± 0.17	0.58 ± 0.27	0.71 ± 0.29	0.207^a^
AST (U/L)	61.09 ± 38.32	63.43 ± 43.60	88.00 ± 63.33	0.199^a^
ALT (U/L)	100.55 ± 79.23	109.50 ± 62.14	134.89 ± 115.90	0.382^a^
Triglyceride (mg/dL)	143.00 ± 62.45	144.63 ± 72.95	146.95 ± 77.33	0.989^a^
Cholesterol (mg/dL)	174.82 ± 27.48	169.83 ± 31.00	172.00 ± 23.12	0.876^a^
LDL (mg/dL)	114.91 ± 29.85	110.53 ± 28.92	112.79 ± 24.45	0.896^a^
HDL (mg/dL)	44.27 ± 8.37	41.90 ± 9.36	43.53 ± 9.95	0.721^a^
Albumin (g/dL)	4.71 ± 0.32	4.70 ± 0.19	4.72 ± 0.25	0.900^a^
Platelet (*10^3^/uL)	335.27 ± 58.90	289.30 ± 59.28	307.05 ± 70.98	0.122^a^
Insulin (μIU/mL)	26.35 ± 11.50	31.76 ± 31.75	33.57 ± 23.77	0.771^a^
Fasting plasma glucose (mg/dL)	89.82 ± 8.96	96.37 ± 14.58	92.89 ± 6.77	0.254^a^
Fibroscan—CAP (dB/m)	281.18 ± 38.57	302.07 ± 30.07	300.89 ± 40.07	0.225^a^
FAST score	0.41 ± 0.17	0.45 ± 0.15	0.52 ± 0.47	0.145^a^
FIB-4 score	0.26 ± 0.08	0.27 ± 0.11	0.40 ± 0.31	0.073 ^a^
Fibroscan—LSM (kPa)	6.12 ± 1.37	6.55 ± 1.51	6.65 ± 1.82	0.731^a^
Interleukin 6 (pg/mL)	3.13 ± 1.33	3.27 ± 2.05	3.89 ± 2.04	0.471^a^
P1NP (ng/ml)	439.19 ± 332.31	450.60 ± 272.09	319.11 ± 202.15	0.223^a^
Osteocalcin (ng/ml)	90.81 ± 55.27	90.20 ± 51.49	69.94 ± 33.78	0.307^a^
P1NP/Osteocalcin	4.36 ± 1.57	5.02 ± 1.96	4.67 ± 1.67	0.263^a^
M2BPGi (ng/ml)	0.74 ± 0.32	0.81 ± 0.66	0.83 ± 0.45	0.901^a^

*P1NP, Procollagen type 1 N-terminal propeptide; M2BPGI, Mac-2 binding glycosylation isomer; FAST, Fibroscan-AST. Values are n (percentage) or mean ± standard deviation*.

† *Ultrasonography*.

a *One-way ANOVA test*.

**Table 2:** Comparison of clinical characteristics, laboratory results, and biomarkers released during liver inflammation and fibrogenesis in patients who had positive and non-positive FAST scores [comparison between patients with steatohepatitis (early fibrosis) and patients with non-steatohepatitis].

	**Group of FAST-positive**	**Group of FAST-negative**	***P*** **-value**
	***N*** **(percentage) or mean** **±SD**		
Patient number	46 (76.7%)	14 (23.3%)	
Age (years)	13.54 ± 2.65	14.69 ± 3.17	0.223^a^
Male/female	39 (84.8%) / 7 (15.2%)	10 (71.4%) / 4 (28.6%)	0.284^b^
BMI z score	3.41 ± 1.30	3.23 ± 1.56	0.470^a^
Total bilirubin (mg/dL)	0.63 ± 0.28	0.58 ± 0.22	0.356^a^
AST (U/L)	80.96 ± 53.61	37.36 ± 6.70	**0.006** ^a^
ALT (U/L)	133.78 ± 74.86	57.14 ± 22.40	**<0.001** ^a^
Triglyceride (mg/dL)	137.80 ± 64.44	168.93 ± 89.37	0.188^a^
Cholesterol (mg/dL)	169.09 ± 27.92	179.14 ± 26.42	0.514^a^
LDL (mg/dL)	109.41 ± 27.58	120.71 ± 25.56	0.516^a^
HDL (mg/dL)	43.63 ± 9.87	40.29 ± 6.65	0.298^a^
Albumin (g/dL)	4.74 ± 0.22	4.64 ± 0.33	0.214^a^
Platelet (*10^3^/uL)	303.89 ± 67.49	301.57 ± 55.21	0.708^a^
Insulin (μIU/mL)	34.48 ± 29.03	21.03 ± 9.73	**0.018** ^a^
Fasting plasma glucose (mg/dL)	94.83 ± 12.45	91.57 ± 9.13	0.475^a^
**Ultrasonography**			
Mild	5 (10.8%)	5 (35.7%)	**0.033** ^b^
Moderate	24 (52.2%)	7 (50.0%)	
Severe	17 (37.0%)	2 (14.3%)	
Fibroscan—CAP (dB/m)	303.78 ± 34.64	278.43 ± 31.47	**0.033** ^a^
FAST score	0.52 ± 0.12	0.27 ± 0.08	**<0.001** ^a^
FIB-4 score	0.33 ± 0.22	0.26 ± 0.11	0.145^a^
Fibroscan—LSM (kPa)	6.84 ± 1.89	5.41 ± 0.98	**0.005** ^a^
Interleukin 6 (pg/mL)	3.59 ± 2.11	2.96 ± 1.11	0.271^a^
P1NP (ng/ml)	404.22 ± 239.91	415.59 ± 351.97	0.599^a^
Osteocalcin (ng/ml)	79.37 ± 36.59	98.74 ± 72.92	0.095^a^
P1NP/osteocalcin	4.92 ± 1.75	3.85 ± 1.06	**0.008** ^a^
P1NP/ALP	1.80 ± 1.14	1.67 ± 0.57	**0.003** ^a^
M2BPGi (ng/ml)	0.85 ± 0.61	0.64 ± 0.19	0.202^a^

*P1NP, Procollagen type 1 N-terminal propeptide; M2BPGI, Mac-2 binding glycosylation isomer; FAST, Fibroscan-AST. Values are n (percentage) or mean ± standard deviation*.

a *T-test*.

b *Mann–Whitney test. The bold values indicates significant p values less than 0.05*.

**Table 3:** Comparison of the clinical characteristics, laboratory results, and biomarkers released during liver inflammation and fibrogenesis in patients who had positive and non-positive FAST scores (comparison between patients with advanced fibrosis and with non-advanced fibrosis).

	**Group of LSM-positive**	**Group of LSM-negative**	***P*** **-value**
	***N*** **(percentage) or mean** **±SD**		
Patient number	7 (11.7%)	53 (88.3%)	
Age (years)	13.92 ± 4.35	13.83 ± 2.66	0.936^a^
Male/female	6 (85.7%)/1 (14.3%)	43 (81.1%)/10 (18.9%)	0.772^b^
BMI z score	4.01 ± 1.82	3.29 ± 1.28	0.343^a^
Total bilirubin (mg/dL)	0.70 ± 0.25	0.61 ± 0.27	0.410^a^
AST (U/L)	148.29 ± 69.62	60.55 ± 37.63	**<0.001** ^a^
ALT (U/L)	177.71 ± 95.24	107.74 ± 67.52	**0.017** ^a^
Triglyceride (mg/dL)	111.29 ± 38.23	149.53 ± 73.81	0.051^**a**^
Cholesterol (mg/dL)	155.14 ± 36.01	173.58 ± 26.08	0.232^a^
LDL (mg/dL)	95.71 ± 33.35	114.21 ± 26.06	0.2011^a^
HDL (mg/dL)	43.71 ± 14.51	42.74 ± 8.56	0.796^a^
Albumin (g/dL)	4.71 ± 0.29	4.72 ± 0.25	0.995^a^
Platelet (*10^3^/uL)	276.00 ± 40.40	306.96 ± 66.36	0.110^a^
Insulin (μIU/mL)	44.97 ± 51.74	32.57 ± 29.41	0.148^a^
Fasting plasma glucose (mg/dL)	100.14 ± 23.82	93.26 ± 9.28	0.148^a^
**Ultrasonography**			
Mild	0 (0.0%)	10 (18.9%)	0.584^b^
Moderate	4 (57.1%)	27 (50.9%)	
Severe	3 (42.9%)	16 (30.2%)	
Fibroscan—CAP (dB/m)	288.43 ± 45.21	299.11 ± 34.19	0.566^a^
FAST score	0.71 ± 0.11	0.43 ± 0.13	**<0.001** ^a^
FIB-4 score	0.62 ± 0.43	0.27 ± 0.10	**<0.001** ^a^
Fibroscan—LSM (kPa)	10.30 ± 2.25	6.00 ± 0.98	**<0.001** ^a^
Interleukin 6 (pg/mL)	5.31 ± 3.99	3.19 ± 1.37	**0.005** ^a^
P1NP^a^ (ng/ml)	322.44 ± 354.42	418.02 ± 255.40	0.513^a^
Osteocalcin (ng/ml)	61.06 ± 51.60	86.91 ± 46.67	0.246^a^
P1NP/Osteocalcin	4.60 ± 1.71	4.68 ± 1.68	0.915^a^
M2BPGi (ng/ml)	1.47 ± 1.24	0.72 ± 0.30	**<0.001** ^a^

*P1NP, Procollagen type 1 N-terminal propeptide; M2BPGI, Mac-2 binding glycosylation isomer; FAST, Fibroscan-AST. Values are n (percentage) or mean ± standard deviation*.

a *T-test*.

b *Mann–Whitney test. The bold values indicates significant p values less than 0.05*.

All of the above statistical analysis were conducted using SPSS version 27 (IBM Corporation, Armonk, NY, USA). A *p*-value <0.05 was considered as statistically significant.

## Results

### Patient Characteristics

The mean age of the patients (*n* = 60) was 13.84 years, and the majority of the patients were male (81.7%) (Table 4). The mean value of BMI evaluated by the Z score was 3.37, indicating obesity (25). Six patients with insulin resistance assessed by a homeostatic model assessment for insulin resistance value, accounted for 10.0% of all patients. Two patients were diagnosed with type 2 diabetes. No patient had bilirubin level higher than normal; additionally, ALT elevation (mean value: 70.78 U/L) was more prominent than AST elevation (mean value: 115.90 U/L). Although the test was performed after fasting, the triglyceride level was 145.07 mg/dL, which was high when compared to the normal values considering the age. The cholesterol and low-density lipoprotein levels were 171.43 and 112.05 mg/dL, respectively.

**Table 4:** Baseline clinical characteristics of the patients.

	***N*** **(percentage) or mean ±SD**
**Demographics**	
Total patient number	60
Age (years)	13.84 ± 2.83
Male	49 (81.7%)
Female	11 (18.3%)
BMI z score	3.37 ± 1.34
**Metabolic**	
HOMA-IR score	1.72 ± 1.41
Insulin resistance	6 (10.0%)
Diabetes	2 (3.3%)
**Laboratory test**	
Total bilirubin (mg/dL)	0.62 ± 0.26
AST (U/L)	70.78 ± 50.05
ALT (U/L)	115.90 ± 73.23
Triglyceride (mg/dL)	145.07 ± 70.84
Cholesterol (mg/dL)	171.43 ± 27.46
LDL (mg/dL)	112.05 ± 27.11
HDL (mg/dL)	42.85 ± 9.20
Albumin (g/dL)	4.72 ± 0.25
Platelet (*10^3^/uL)	303.35 ± 63.86
Insulin (μIU/mL)	34.01 ± 32.14
Fasting plasma glucose (mg/dL)	94.07 ± 11.67
**Ultrasonography**	
Mild	10 (16.7%)
Moderate	31 (51.7%)
Severe	19 (31.7%)
**Steatosis**	
CAP, dB/m (fibroscan)	297.87 ± 35.06
**Steatohepatitis**	
FAST score (cut-off >0.35)	0.47 ± 0.16
**Fibrosis**	
FIB4 score (cut-off >3.25)	0.31 ± 0.20
LSM, kPa (fibroscan)	6.57 ± 1.83

*Values are n (percentage) or mean ± standard deviation*.

The degree of fatty liver confirmed by ultrasonography was 31/60 (51.7%) in the moderate stage, accounting for more than half the cases. The quantified steatosis, CAP obtained through fibroscan was confirmed with the mean value of 297.87 dB/m. The FAST score for evaluating steatohepatitis had the mean value of 0.47. LSM, which evaluates fibrosis, showed the mean value of 6.57 kPa.

### Biomarker to Evaluate the Stage of Steatosis

The clinical characteristics, laboratory results, and biomarker values were compared by dividing the patients into three groups according to their grade (mild, moderate, and severe) of fatty liver on ultrasonography (Table 1). Among 60 patients, 10 patients (16.7%) were identified as having mild fatty liver, 31 patients (51.7%) were identified as having moderate fatty liver, and 19 patients (31.7%) were identified as having severe fatty liver on ultrasonography. No statistically significant differences were observed between the three groups in clinical characteristics, laboratory results, scores, and biomarkers. However, when looking at the mean value of each group, there are items worth interpreting, although there is no statistical significance. In the laboratory results, the mean levels of AST, ALT, triglyceride, and insulin were observed to increase as the fatty liver grade increases. It was expected that the CAP test result would show a statistically significant difference between groups as it had a linear relationship with the degree of fatty liver evaluated by ultrasonography; however, it did not show a significant difference (*p*-value: 0.225).

### Biomarkers to Evaluate the Stage of Steatohepatitis (in the Process of Fibrogenesis)

The clinical characteristics, laboratory results, and biomarker values were compared by dividing the patients into two groups: FAST-positive group (FAST score of 0.35 or higher) and FAST-negative group (FAST score of <0.35) (Table 2). Among 60 patients, 47 patients (76.7%) were identified as the FAST-positive group and 14 patients (23.3%) were identified as the FAST-negative group. The mean value of the FAST score of FAST-positive group was 0.52 and that of FAST-negative group was 0.27. No significant differences were observed between the two groups in clinical characteristics, such as age, sex, and BMI z score. In the laboratory results, the AST, ALT, and insulin levels showed a significant difference (*p*-value 0.006, <0.001, and 0.018, respectively) between the two groups, and all three values showed significantly higher values in the FAST-positive group (AST: 80.96 vs. 37.36, ALT: 133.78 vs. 57.14, and insulin: 34.48 vs. 21.03).

There was also a statistically significant difference in the ratio of mild, moderate, and severe fatty liver confirmed by ultrasonography. In the FAST-positive group, the percentage of patients in the severe status was 37% and was significantly higher than 14.3% in the negative group. CAP and LSM showed a significant difference between the two groups with *p*-values of 0.033 and 0.005, respectively; additionally, the FAST-positive group showed a higher mean value (CAP: 303.78 vs. 278.43, LSM: 6.84 vs. 5.41). Among the biomarkers (IL-6, P1NP, osteocalcin, and M2BPGi), P1NP/osteocalcin ratio and P1NP/ALP ratio (corrected ECM product) showed significant differences with *p*-values of 0.008 and 0.003, respectively. Pearson's correlation between osteocalcin and ALP showed a significant correlation with a *p*-value < 0.001.

A logistic regression analysis was performed on the basis of the results of Table 2, the P1NP/osteocalcin ratio and ALT were evaluated as suitable factors in the regression formula with statistical significance (Table 5A). For the P1NP/osteocalcin ratio, the odds ratio was 1.850, with a *p*-value of 0.018; additionally, for ALT, the odds ratio was 1.078, with a *p*-value of 0.022. Based on these results, whether the P1NP/osteocalcin ratio had a diagnostic capability in predicting steatohepatitis (early fibrosis) or not was evaluated using the ROC curve (Figure 2). Even the P1NP/osteocalcin ratio alone, with AUROC of 0.782, *p*-value of 0.002, and cut-off value of 3.54 or higher, had a diagnostic capability to evaluate steatohepatitis (early fibrosis); however, the diagnostic capability for assessing the steatohepatitis was higher when the ratio was multiplied by ALT. The AUROC of P1NP/osteocalcin ratio^*^ALT value was 0.939 and the *p*-value was <0.001. The cut-off value was 305.38. P1NP/ALP ratio evaluation through AUROC curve also showed a diagnostic capability, with AUROC of 0.788, *p*-value of 0.001, and cut-off value of 1.46 or higher. Similarly, the P1NP/ALP ratio showed better diagnostic capability when the ratio was multiplied by ALT, with AUROC of 0.894, *p*-value of <0.001, and cut-off value of 119.08 or higher.

**Table 5A:** Univariate and logistic multivariate analyses of the association between steatohepatitis (early fibrosis) assessed by the FAST score and other factors.

**Parameter**	**Univariate analysis**			**Multivariate analysis**		
	***P*** **-value**	**Odds ratio**	**95% CI**	***P*** **-value**	**Odds ratio**	**95% CI**
Insulin (μIU/mL)	0.758	1.010	0.947–1.077			
Ratio (P1NP/Osteocalcin)	0.023	1.724	0.608–1.890	0.018	1.850	0.682–5.018
AST (U/L)	0.118	1.546	0.896–2.668			
ALT (U/L)	0.028	1.066	1.007–1.129	0.022	1.078	1.013–1.147

*Multivariate analysis was performed by selecting variables with a p value < 0.1 in univariate analysis*.

### Biomarker to Evaluate the Stage of Advanced Fibrosis

The clinical characteristics, laboratory results, and biomarker values were compared by dividing the patients into two groups with LSM-positive group (LSM of 8.2 kPa or higher), and LSM-negative group (LSM of <8.2 kPa) (Table 3). Among 60 patients, 7 patients (11.7%) were identified as the LSM-positive group and 53 patients (88.3%) were identified as the LSM-negative group. The mean value of LSM of the LSM-positive group was 10.30 and that of the LSM-negative group was 6.00. No significant differences were observed between the two groups in clinical characteristics, such as age, sex, and BMI z score. In the laboratory results, the AST and ALT levels showed a significant difference (*p*-value <0.001 and 0.017, respectively) between the two groups, and the two values showed significantly higher values in the LSM-positive group (AST: 148.29 vs. 60.55 and ALT: 177.71 vs. 107.74).

The FAST score and fibrosis-4 score showed a significant difference between the two groups with both *p*-values of <0.001; additionally, the LSM-positive group showed higher mean value (FAST score: 0.71 vs. 0.43, fibrosis-4 score: 0.62 vs. 0.27). Among the biomarkers (IL-6, P1NP, osteocalcin, and M2BPGi), only IL-6 and M2BPGi showed significant differences with *p*-values of 0.005 and <0.001, respectively.

A logistic regression analysis was performed based on the results of Table 3; the IL6 and AST were evaluated as suitable factors in the regression formula with statistical significance (Table 5B). For the IL-6, the odds ratio was 1.844, with a *p*-value of 0.010, and for ALT, the odds ratio was 1.010, with a *p*-value of 0.045. Based on these results, whether the IL-6 has a diagnostic capability in predicting advanced fibrosis was evaluated using the ROC curve (Figure 3). The AUROC of IL-6 as 0.719, *p*-value of 0.037, and cut-off value of 5.25 or higher had a diagnostic capability to evaluate advanced fibrosis; however, the diagnostic capability for assessing advanced fibrosis was higher when the ratio was multiplied by AST. The AUROC of IL-6^*^ AST value was 0.821 (*p* = 0.002) and the cut-off value was 228.15.

**Table 5B:** Univariate and multivariate analyses of the association between advanced fibrosis assessed by liver stiffness measurement (kPa) and other factors.

**Parameter**	**Univariate analysis**			**Multivariate analysis**		
	***P*** **-value**	**Odds ratio**	**95% CI**	***P*** **-value**	**Odds ratio**	**95% CI**
Triglyceride (mg/dL)	0.346	0.988	0.964–1.013			
Interleukin 6 (pg/mL)	0.084	2.322	0.892–6.045	0.010	1.844	1.161–2.928
AST (U/L)	0.633	1.005	0.984–1.026	0.045	1.010	1.000–1.020
ALT (U/L)	0.871	1.003	0.970–1.037			
M2BPGi (ng/ml)	0.172	32.414	0.221–1.153			

*Multivariate analysis was performed by selecting variables with p-values < 0.1 in univariate analysis*.

M2BPGi, which has already proven its diagnostic value for advanced fibrosis or cirrhosis in chronic liver disease, was evaluated to check whether it can be utilized in pediatric NAFLD. LSM (kPa) and M2BPGi showed a significant linear relationship in the Pearson correlation analysis (Pearson correlation coefficient = 0.382 with a *p*-value of 0.003) (Figure 4A). The diagnostic capability evaluation of advanced fibrosis with AUROC also showed an acceptable result (AUROC = 0.742 with a *p*-value of 0.022) (Figure 4B).

## Discussion

**Figure 5:**
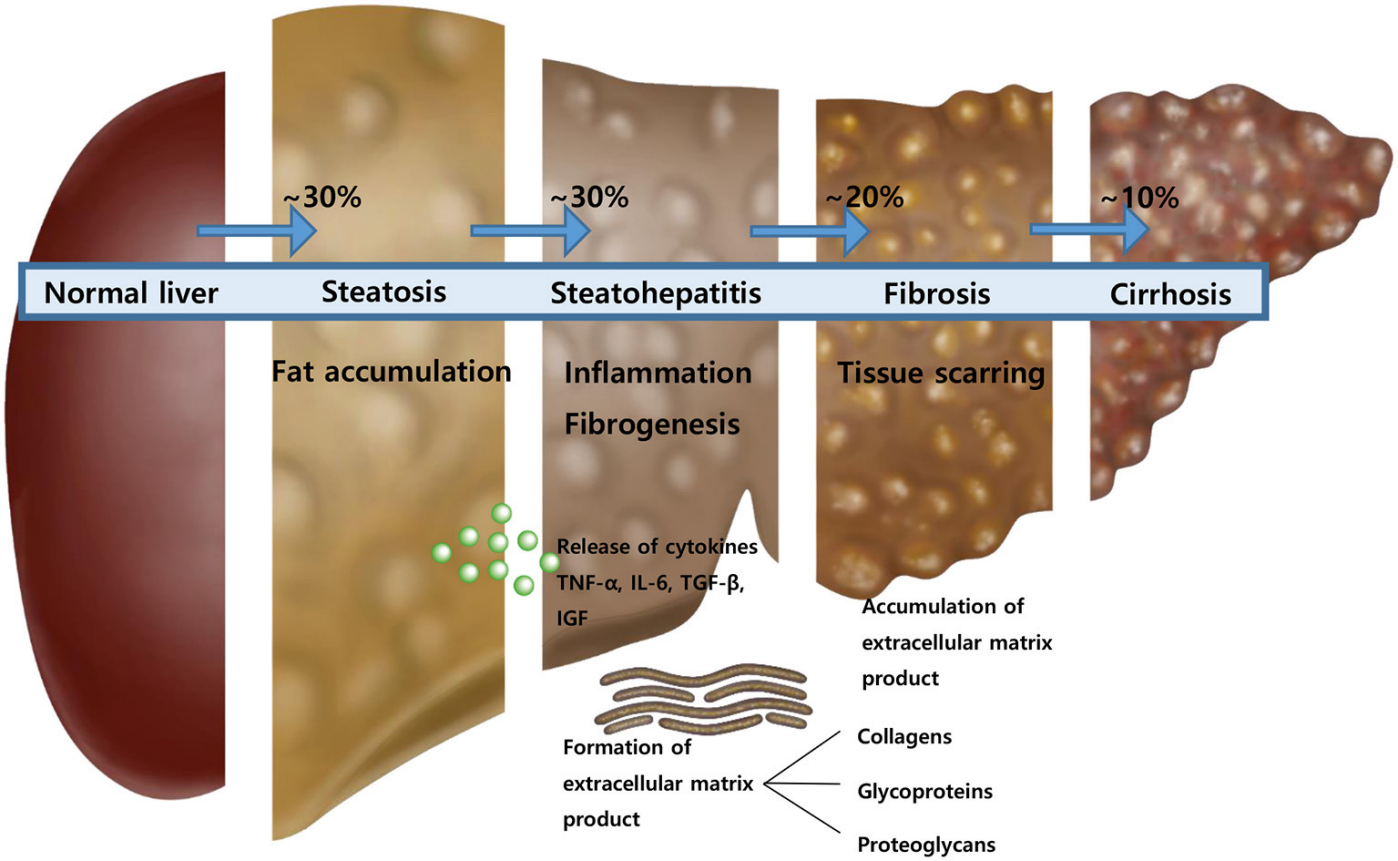
An illustration showing the course of disease exacerbation of non-alcoholic fatty liver.

We prospectively studied the diagnostic capability of biomarkers for predicting each stage of NAFLD in children and adolescents with fatty liver disease (Figure 5). Biomarkers could be used to determine the status after treatment because NAFLD may not be completely resolved even if AST and ALT are normalized. In a series of processes from steatosis to steatohepatitis to fibrosis and to cirrhosis, inflammatory cytokines and ECM products are formed. This study evaluated each stage of NAFLD by measuring these substances.

Our major findings were as follows. First, previous studies evaluated that amino-terminal propeptide of type III procollagen (PIIINP) could be used as a biomarker, but in this study, P1NP, a popular test, as it is relatively convenient to measure and is frequently performed in osteoporosis patients, was evaluated as a biomarker and its diagnostic capability was confirmed. Second, previous studies evaluated that interleukin such as IL-8 and IL-18 could be secreted during proinflammatory or inflammatory processes and used as biomarkers, but this study evaluated fibrosis stage with IL-6, which is secreted during the regeneration process after an inflammatory response. Third, this study initially evaluated that M2BPGi, a known biomarker to predict advanced fibrosis or cirrhotic changes in chronic liver disease, has the diagnostic capability in pediatric NAFLD. Taken together, the measurements of P1NP, IL-6 or M2BPGi along with the basic chemistry tests would help determine the stage of NAFLD they correspond to at the time of initial diagnosis and predict responsiveness after the treatment.

Our first major finding is the usefulness of the P1NP/osteocalcin ratio or P1NP/ALP ratio to evaluate steatohepatitis (in the process of fibrogenesis). In the case of PIIINP, a procollagen known as a biomarker, it is difficult to measure because it is not a widely used test, so we tried to evaluate other ECM products. Among various ECM products, P1NP, one of procollagen, was selected as an evaluation biomarker because it is widely used to evaluate the treatment response of osteoporosis patients. The advantage of P1NP is that results can be obtained by collecting blood at most hospitals in Korea, and the price is lower than that of other collagens (about 33.1 U.S. $ without insurance adjustment). Additionally, the results can be obtained within 2 h on the same day.

Some other studies have also suggested that P1NP is formed in the liver during fibrogenesis (26–28). These are recent studies and includes adult patients with NAFLD. An animal using mice with bile duct ligation-induced fibrosis also confirmed P1NP formation during fibrogenesis (29). We think that inflammation (steatohepatitis) and fibrogenesis (early fibrosis) are mixed because they are a series of processes. As a result of our study, P1NP was significantly evaluated on the basis of the FAST score (steatohepatitis); thus, we believe that P1NP predicts the stage of inflammation with early fibrosis rather than advanced fibrosis. However, as the normal levels of P1NP in children and adolescent patients are different because it is bone-related factor, we planned to correct P1NP using osteocalcin or ALP. As can be seen from the results, the statistical significance could not be confirmed with P1NP alone; however, the statistical significance was confirmed after correction by osteocalcin or ALP.

Hepatocyte injury in hepatitis results in the leakage of enzymes into the circulation. Therefore, both AST and ALT act as important liver function tests that are elevated in patients with NAFLD. However, ALT is more specific for liver damage since it is found primarily in the liver; however, the AST is also found in several other organs. Therefore, in our study, both AST and ALT were statistically significant in an early liver injury situation; nevertheless, the *p*-value of ALT (<0.001) was lower than the *p*-value of AST (0.006), and ALT was selected in the logistic regression analysis in Table 5A. Therefore, based on the results of the logistic regression analysis, it was confirmed that the diagnostic capability using the AUROC curve was further increased when the above-mentioned P1NP/osteocalcin ratio and ALT were multiplied (Figure 3).

Our second major finding is the usefulness of the IL-6, an inflammatory cytokine, to evaluate advanced fibrosis (30). IL-6 is involved in immune, hematopoietic, and inflammatory responses by acting on T cells, hepatocytes, and hematopoietic stem cells. IL-6 increases rapidly even during infections, inflammation, and trauma, and shows high serum concentrations in rheumatoid arthritis and autoimmune diseases. We initially checked whether there are other infections of inflammatory signs currently through the questionnaire. We initially hypothesized that IL-6 might be involved in the early inflammatory stage of NAFLD because it is an inflammatory cytokine. However, herein, it was meaningfully evaluated in the analysis of the advanced stage of fibrosis. Considering the reasons for the result, other studies demonstrated that a strong activation of the transcription factors has been described through animal studies using mice, resulting in the enhanced transcription of their target genes after the increase in the IL-6 serum levels (31, 32). Through these processes, IL-6 maintains adequate levels of anti-apoptotic factors and is an important mediator of liver regeneration (31–33). We believe that patients with elevated IL-6 levels in this study are in the process of regeneration as a late response after an injury. Therefore, it is considered to be highly measured in patients with advanced fibrosis who have had inflammation for a long time. As a result, the value of IL-6 was significantly higher in patients who were LSM-positive (Table 3). The advantage of IL-6 is that the results can be obtained by collecting blood at most hospitals in Korea; additionally, the price is low compared to other inflammatory cytokines (~72.8 USD without insurance adjustment). Moreover, the results can be obtained within 1 week. In the case of TNF, it takes a month to obtain the test results and the cost is three times that of IL-6.

The fact that AST showed more significant difference than ALT in patients with advanced fibrosis can be explained in the same context. Several studies have been conducted on the AST/ALT ratio in patients with NAFLD. Although this ratio is used to distinguish fibrosis from alcoholic fatty liver, it is also used to differentiate between early and advanced fibroses. The AST/ALT ratio, with a value >0.8, is considered to be associated with advanced fibrosis (34, 35). Therefore, in our study, it seems that AST, not ALT, was evaluated as a significant factor in the logistic regression analysis performed on the basis of LSM positivity. Therefore, based on the results of a logistic regression analysis, it was confirmed that the diagnostic capability with the AUROC curve was further increased when the above-mentioned IL-6 and AST were multiplied (Figure 4).

Our third major finding is that M2BPGi has a diagnostic capability even in pediatric NAFLD. M2BPGi has proven to be an appropriate biomarker to evaluate advanced fibrosis or cirrhotic changes in chronic liver disease. Regarding adults, there are various articles evaluating the diagnostic capability of patients with NAFLD (36–39). However, regarding children, there are only articles evaluating biliary atresia or multiple diseases together (40–43). It is of great significance that we evaluated the diagnostic capability of M2BPGi for assessing the advanced fibrosis status in pediatric patients with NAFLD for the first time. Although the AUROC value did not exceed 0.8, it showed a cut-off value (1.35 ng/ml) similar to that of the F3 state (1.57 ng/ml) in the adult study with a statistically significant *p*-value (38). Therefore, we concluded that M2BPGi can be applied to diagnose an advanced fibrosis status in pediatric NAFLD.

To correct the errors that occur when dividing by only one criterion FAST score or LSM, an analysis of subgroups was also conducted using both FAST score and LSM. Supplementary Figure 1 shows the results of biomarker comparisons between the group with both negative FAST scores and LSM, and the group with only positive FAST scores (LSM is negative). In this subgroup, the group with only positive FAST score can be considered specific for steatohepatitis (early fibrosis). As can be seen in the figure, only the P1NP/osteocalcin ratio was statistically significantly evaluated (*p*-value = 0.043). Supplementary Figure 2 shows the biomarker results compared by dividing the group with both positive FAST scores and LSM, and the group with only positive FAST scores (LSM is negative). In this subgroup, the group with both positive LSM and FAST scores can be considered specific for advanced fibrosis. As can be seen in the figure, M2BPGi and IL-6 show a statistically significant difference for these subgroups (*p*-value = 0.002, 0.001, respectively).

The major limitation of this study is that liver biopsy was not performed and not directly compared to the histologic grade because the most accurate NAFLD diagnosis method is still a biopsy. However, the importance of non-invasive diagnoses is emerging in adults; additionally, non-invasiveness is particularly important in children. We conducted the study on the basis of recent papers with high reliability in the criteria for determining positive and negative results. Another limitation of this study is that information on pubertal status was not collected. Since previous studies have found that steatosis, portal inflammation, and fibrosis are less severe during and after puberty than before puberty in subjects with NAFLD, a possible association between puberty status and NAFLD should have considered.

We believe that by evaluating the stage of NAFLD, a more systematic approach for MAFLD and appropriate treatment intervention will be possible. Appropriate treatment at each stage can be suggested by utilizing dietary modifications, exercise, and medications.

## Conclusion

In conclusion, non-invasive biomarkers can be used to predict each stage of NAFLD in children. The value of P1NP/osteocalcin or ALP ratio^*^ ALT has a diagnostic capability to assess the stage of steatohepatitis or in the process of fibrogenesis of NAFLD. The value of IL-6 ^*^ AST has a diagnostic capability to assess the stage of advanced fibrosis of NAFLD. M2BPGi, a known biomarker to predict advanced fibrosis or cirrhotic changes in chronic liver disease, has shown a diagnostic capability even in pediatric NAFLD. Our findings suggest that measuring P1NP, IL-6 or M2BPGi along with the basic chemistry tests would help determine the stage of NAFLD they correspond to at the time of initial diagnosis and predict responsiveness after the treatment. A more systematic approach for MAFLD and appropriate treatment intervention will be possible by evaluating the stage of NAFLD.

## Data Availability Statement

The datasets used and analyzed during the current study are available from the corresponding author on reasonable request.

## Ethics Statement

The studies involving human participants were reviewed and approved by Name of the Ethics Committee and reference: Samsung Medical Center IRB File No.: 2021-02-121. Written informed consent to participate in this study was provided by the participants' legal guardian/next of kin.

## Author Contributions

MK and YC: conception or design. YK and EK: acquisition, analysis, or interpretation of data. YK and MK: drafting the work or revising and final approval of the manuscript. All authors read and approved the manuscript.

## Conflict of Interest

The authors declare that the research was conducted in the absence of any commercial or financial relationships that could be construed as a potential conflict of interest.

## Publisher's Note

All claims expressed in this article are solely those of the authors and do not necessarily represent those of their affiliated organizations, or those of the publisher, the editors and the reviewers. Any product that may be evaluated in this article, or claim that may be made by its manufacturer, is not guaranteed or endorsed by the publisher.
