# Supplementary material for: Stratification by Non-invasive Biomarkers of Non-alcoholic Fatty Liver Disease in Children
Source: Front Pediatr. 2022 Apr 4;10:846273. doi: 10.3389/fped.2022.846273 (PMC9013938; doi:10.3389/fped.2022.846273)
Supplement: Supplementary file 1 [file Data_Sheet_1.pdf]

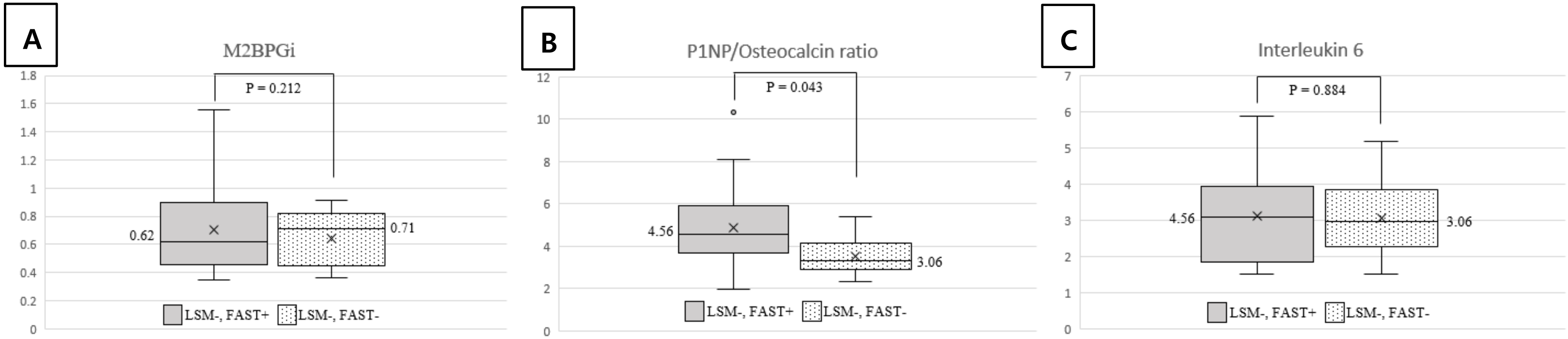

Supplementary Figure 1. (a) Boxplots comparing the median values and interquartile ranges of M2BPGi between a group with negative LSM (kPa) and positive FAST scores and a group with both negative LSM (kPa) and FAST scores. (b) Boxplots comparing the median values and interquartile ranges of the P1NP/Osteocalcin ratio between a group with negative LSM (kPa) and positive FAST scores, and a group with both negative LSM (kPa) and FAST scores. (c) Boxplots comparing the median values and interquartile ranges of interleukin 6 between a group with negative LSM (kPa) and positive FAST scores, and a group with both negative LSM (kPa) and FAST scores.

M2BPGi; Mac-2 binding glycosylation, P1NP; Procollagen type 1 N-terminal propeptide, LSM; Liver stiff measurement, FAST; Fibroscan-AST

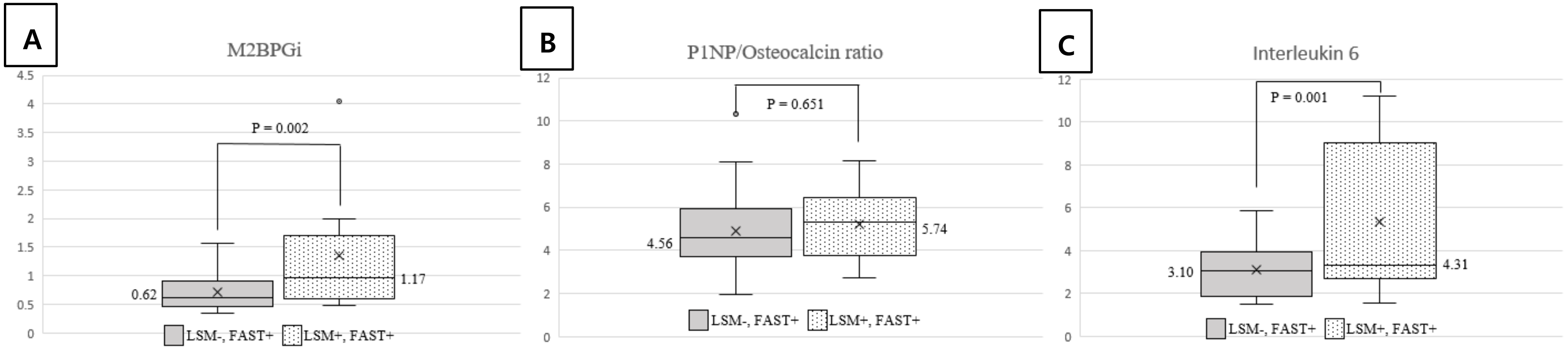

Supplementary Figure 2. (a) Boxplots comparing the median values and interquartile ranges of M2BPGi between a group with negative LSM (kPa) and positive FAST scores, and a group with both positive LSM (kPa) and FAST scores. (b) Boxplots comparing the median values and interquartile ranges of P1NP/Osteocalcin ratio between a group with negative LSM (kPa) and positive FAST scores and a group with both positive LSM (kPa) and FAST scores. (c) Boxplots comparing the median values and interquartile ranges of interleukin 6 between a group with negative LSM (kPa) and positive FAST scores, and a group with both positive LSM (kPa) and FAST scores.

M2BPGi; Mac-2 binding glycosylation, P1NP; Procollagen type 1 N-terminal propeptide, LSM; Liver stiff measurement, FAST; Fibroscan-AST
